# Supplementary material for: Repression of SMAD3 by STAT3 and c-Ski induces conventional dendritic cell differentiation
Source: Life Sci Alliance. 2024 Jul 3;7(9):e201900581. doi: 10.26508/lsa.201900581 (PMC11222659; doi:10.26508/lsa.201900581)
Supplement: Supplementary file 1 [file LSA-2019-00581_TableS1.docx]

**Supplementary Table 1. Primer sequences for quantitative RT-PCR**

| **Gene** | **Sense primer** | **Antisense primer** |
| --- | --- | --- |
|  |  |  |
| *Gapdh* | TGGTGAAGGTCGGTGTGAAC | CCATGTAGTTGAGGTCAATGAAGG |
| *Smad2* | GGAACCTGCATTCTGGTGTT | ACGTTGGAGAGCAAGCCTAA |
| *Smad3* | TTAGGCACCAGCCTGTTTCT | TGGCGATACACCACCTGTTA |
| *Flt3* | TGACGCCCAGTTCACCAAAA | TTTCCTCCGTGCAATTGGGA |
| *Stat3* | AGACCTCTGAGTCTGGGGATG | GGGTATCAGCTCACAGAGTGG |
| *Irf4* | GGGAAACTCCGACAGTGGTT | CCCTTCTCGGAACTTGCCTT |
| *Pu.1* | CTGCGTCTGACCCACGAC | CCAAGTCATCCGATGGAGGG |
| *Gfi1* | GACTCTCAGCTTACCGAGGC | TGCATAGGGCTTGAAAGGCA |
| *Id2* | CACCCTGAACACGGACATCA | AGATCTTGTAGACTTCTTCTTGTGC |
| *Irf2* | AGAGATCGTCACTAACCCGC | TGGTCTCCCCTCTGCGTT |
| *Irf8* | ACGCTGTGCTCTGAACAAGA | GCTCCTCAATCTCTGAGCGG |
| *Batf3* | TTTCGAAGCTGAAGGAGGAGC | TGTGCAAACCAAGGTTCCGA |
| *Relb* | GGTACTGCTAGCCTTGTGGG | AGGTTGGCTTCGGAATGGAG |
| *Flt3L* | TGGAGCCCAAATTCCTCCCT | GGCCACAGTGACTGGGTAAT |
| *c-Ski* | TTGAGGACCTGCAAGCGAAG | CTCAGCGTTGCTCTCACCTC |
| *Csf2ra* | GATGTCAATGGCACGGTCGG | TCGTGACCCGTGGAGTTGAG |
| *Tgfb1* | CGTCAGACATTCGGGAAGCA | GTATCAGTGGGGGTCAGCAG |
| *Tgfb2* | CTAACTTCTGTGCTGGGGCA | GCTGTTCGATCTTGGGCGTA |
| *Tgfb3* | ATCTGTTCCGGGCAGAGTTC | CGAAAGACAGCCATTCAGCG |
| *Cx3cr1* | CCATCTGCTCAGCTCAC | AGATGGTTCCAAAGGCCACA |
| *Clec9a* | TGTTCAGGAGCATGGTGTGT | CCTGCTGCTCCAAGACAAGA |
|  |  |  |
