# Supplementary material for: Repression of SMAD3 by STAT3 and c-Ski induces conventional dendritic cell differentiation
Source: Life Sci Alliance. 2024 Jul 3;7(9):e201900581. doi: 10.26508/lsa.201900581 (PMC11222659; doi:10.26508/lsa.201900581)
Supplement: Supplementary file 2 [file LSA-2019-00581_TableS2.docx]

**Supplementary Table 2. Primer sequences for the *Smad3* proximal promoter region**

| **the *Smad3***  **promoter** | **Sense primer** | **Antisense primer** |
| --- | --- | --- |
|  |  |  |
| -2.0 kb | GATCACGCGTTTGGGTTCAAATTCC  AGCTC | GATCCTCGAGGCAGCAGAAGTTTGGG  TTTC |
|  |  |  |
