# Supplementary material for: Repression of SMAD3 by STAT3 and c-Ski induces conventional dendritic cell differentiation
Source: Life Sci Alliance. 2024 Jul 3;7(9):e201900581. doi: 10.26508/lsa.201900581 (PMC11222659; doi:10.26508/lsa.201900581)
Supplement: Supplementary file 3 [file LSA-2019-00581_TableS3.docx]

**Supplementary Table 3. Primer sequences for ChIP**

| **the *Smad3***  **promoter** | **Sense primer** | **Antisense primer** |
| --- | --- | --- |
|  |  |  |
| -1700 to -1534 | AATGCCAAGTAAGGCACAGG | CTCCCTTCCACTTGCTGCTA |
| -1559 to -1410 | TCTCTAGCAGCAAGTGGAAGG | GCAGCTTGTCAGGGGTTGT |
| -1196 to -1003 | TAGCCTGATAGGGAGGCTGA | CCGGAGAGGACTCGAGAAGT |
| -524 to -372 | GAGCTTTTCTGAACCCCTCA | ACCGGACTCCTGGGGACT |
| -220 to -28 | CTGGGAAGGAGGCTGCAC | AAACTTTGCTGGCCTGGAT |
| -107 to +63 | CAGAGGAGGAGGAGGAGGAG | CGAGACTCCAAGTGGCAGTAG |
|  |  |  |
